# Supplementary figures and images for: Hydrogen sulfide (H2S) coordinates redox balance, carbon metabolism, and mitochondrial bioenergetics to suppress SARS-CoV-2 infection
Source: PLoS Pathog. 2025 May 19;21(5):e1013164. doi: 10.1371/journal.ppat.1013164 (PMC12129340; doi:10.1371/journal.ppat.1013164)

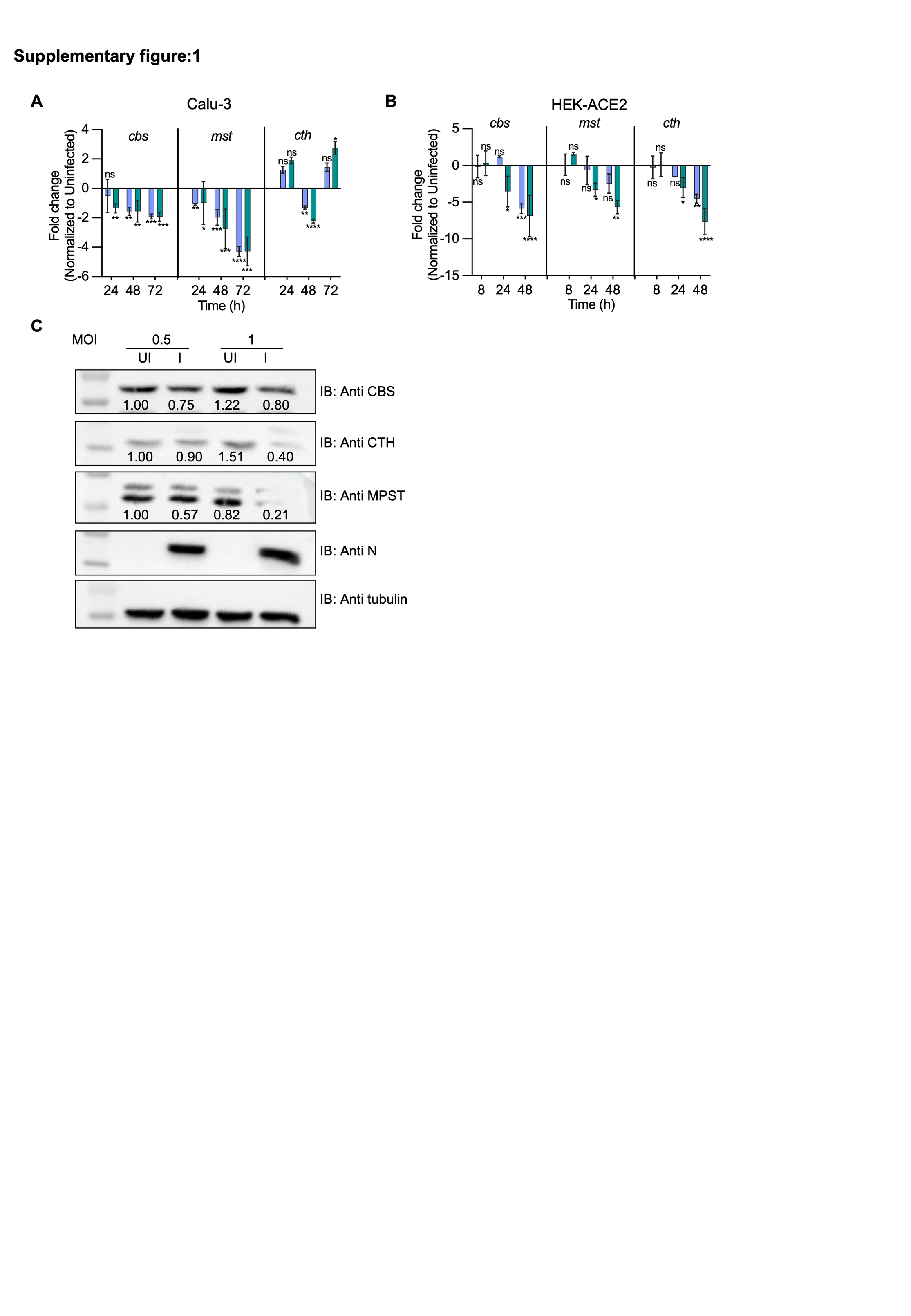

Supplement: S1 Fig — (A) Time-dependent changes in expression of cbs, mst and cth during SARS-CoV-2 (HK variant) replication in Calu-3 cells by RT-qPCR. (B) Time-dependent changes in expression of cbs, mst and cth during SARS-CoV-2-HK replication in HEK-ACE2 cells by RT-qPCR. (C) Protein levels of CBS, CTH and MST during SARS-CoV-2-HK replication in HEK-ACE2 cells, quantified by densitometric analysis using Image Lab software. (TIF) [file ppat.1013164.s001.tif]

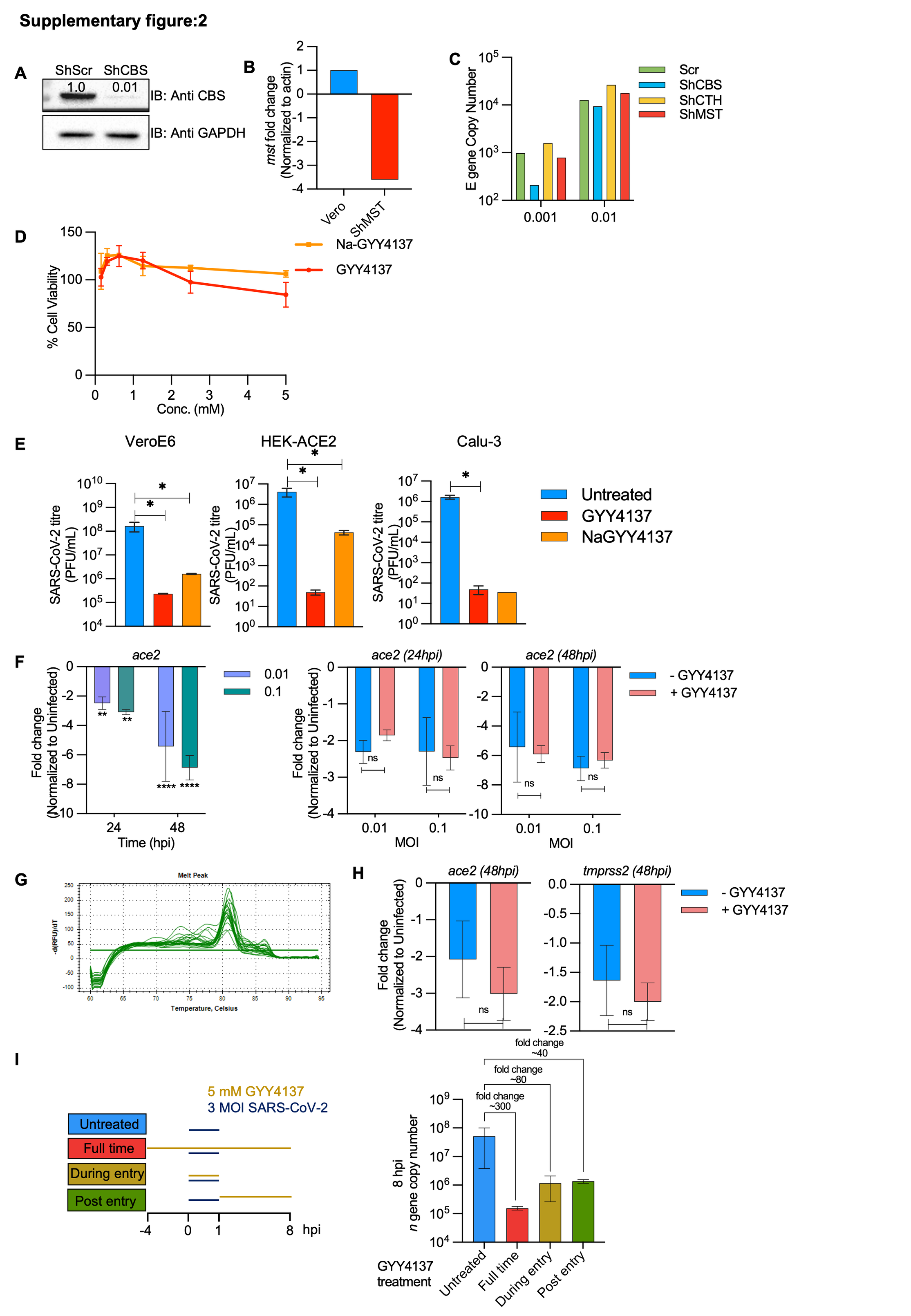

Supplement: S2 Fig — (A) Knockdown confirmation of CBS in VeroE6 cells by western blotting. (B) Knockdown confirmation of mst in VeroE6 cells by RT-qPCR. (C) SARS-CoV-2 viral load in knockdown VeroE6 cells (ShCBS/CTH/MST). (D) Viability of VeroE6 cells in the presence of GYY4137 and Na-GYY4137 at 48 h post treatment by MTT assay. (E) Plaque assay from culture supernatant of different treatment groups. (F) ace2 expression upon SARS-CoV-2 infection and GYY4137 treatment in VeroE6 cells. (G) ace2 and tmprss2 expression in Calu-3 cells, infected with 0.1 MOI SARS-CoV-2 in presence or absence of 5 mM GYY4137 at 48 h p.i. (H) Time of addition experiment of drug GYY4137 in VeroE6 cells. (TIF) [file ppat.1013164.s002.tif]

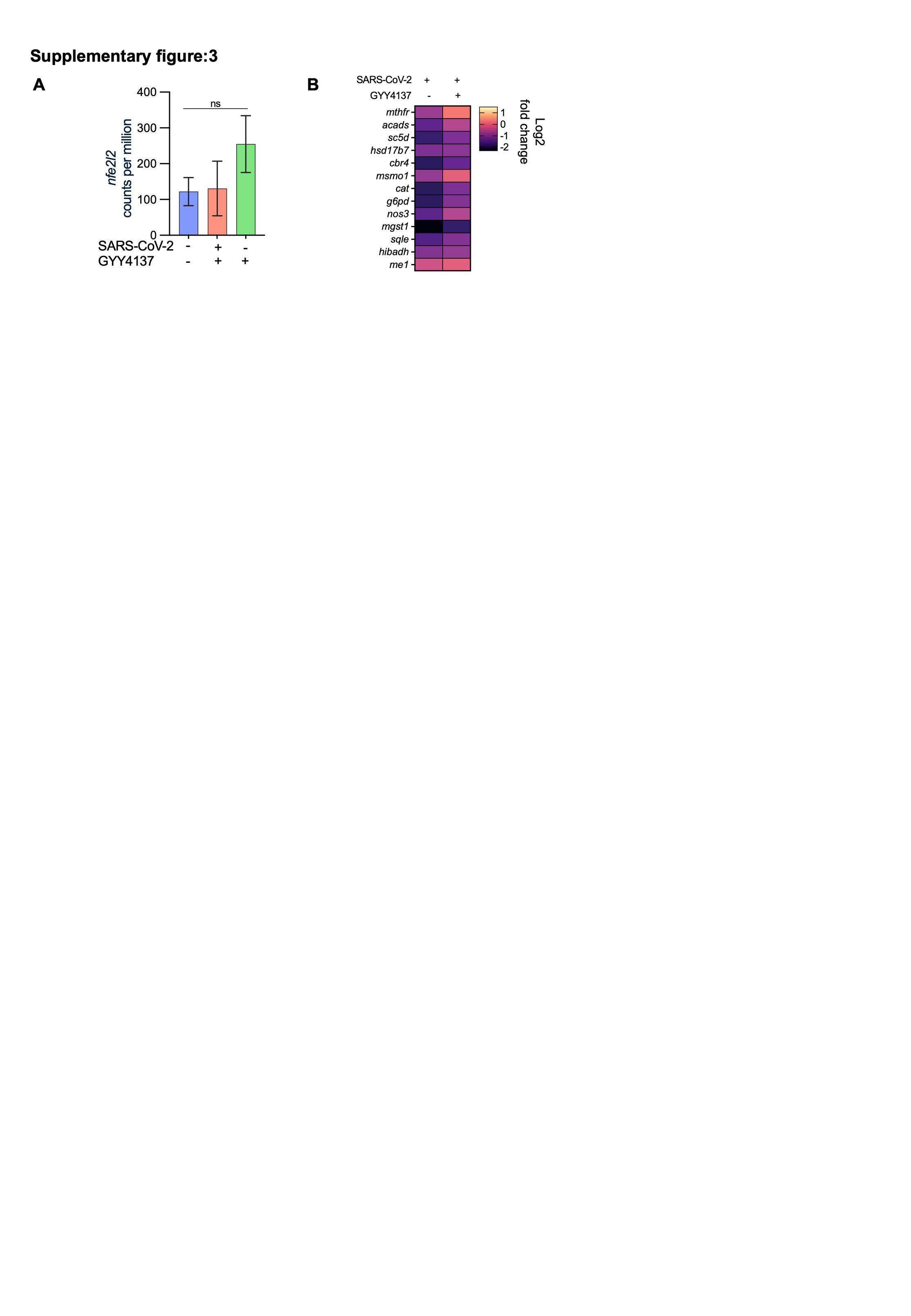

Supplement: S3 Fig — (A) nfe2l2 transcript counts upon SARS-CoV-2 infection in presence or absence of GYY4137 by RNA sequencing. (B) Heat maps of genes associated with oxidoreductase activity. (TIF) [file ppat.1013164.s003.tif]

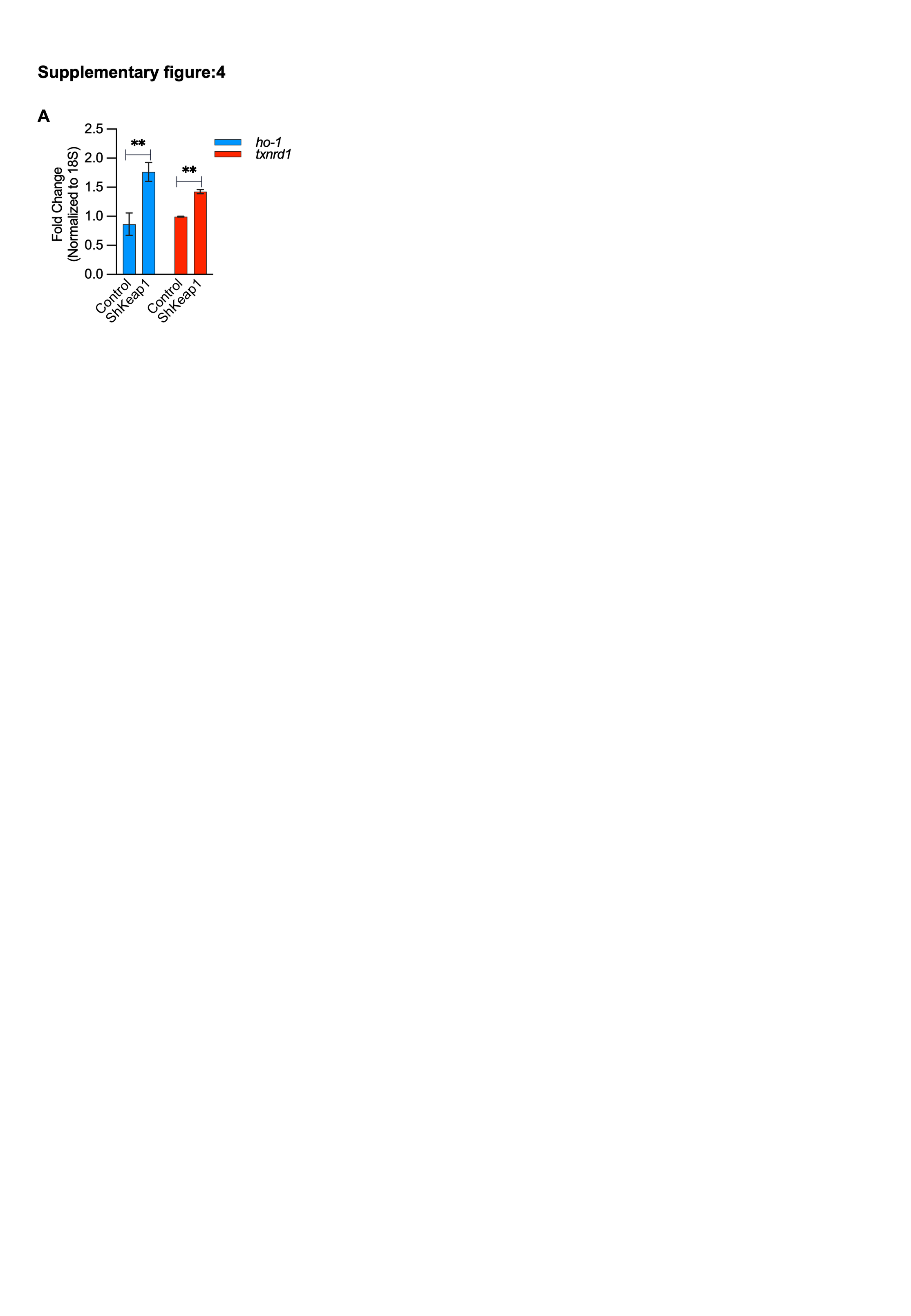

Supplement: S4 Fig — (TIF) [file ppat.1013164.s004.tif]

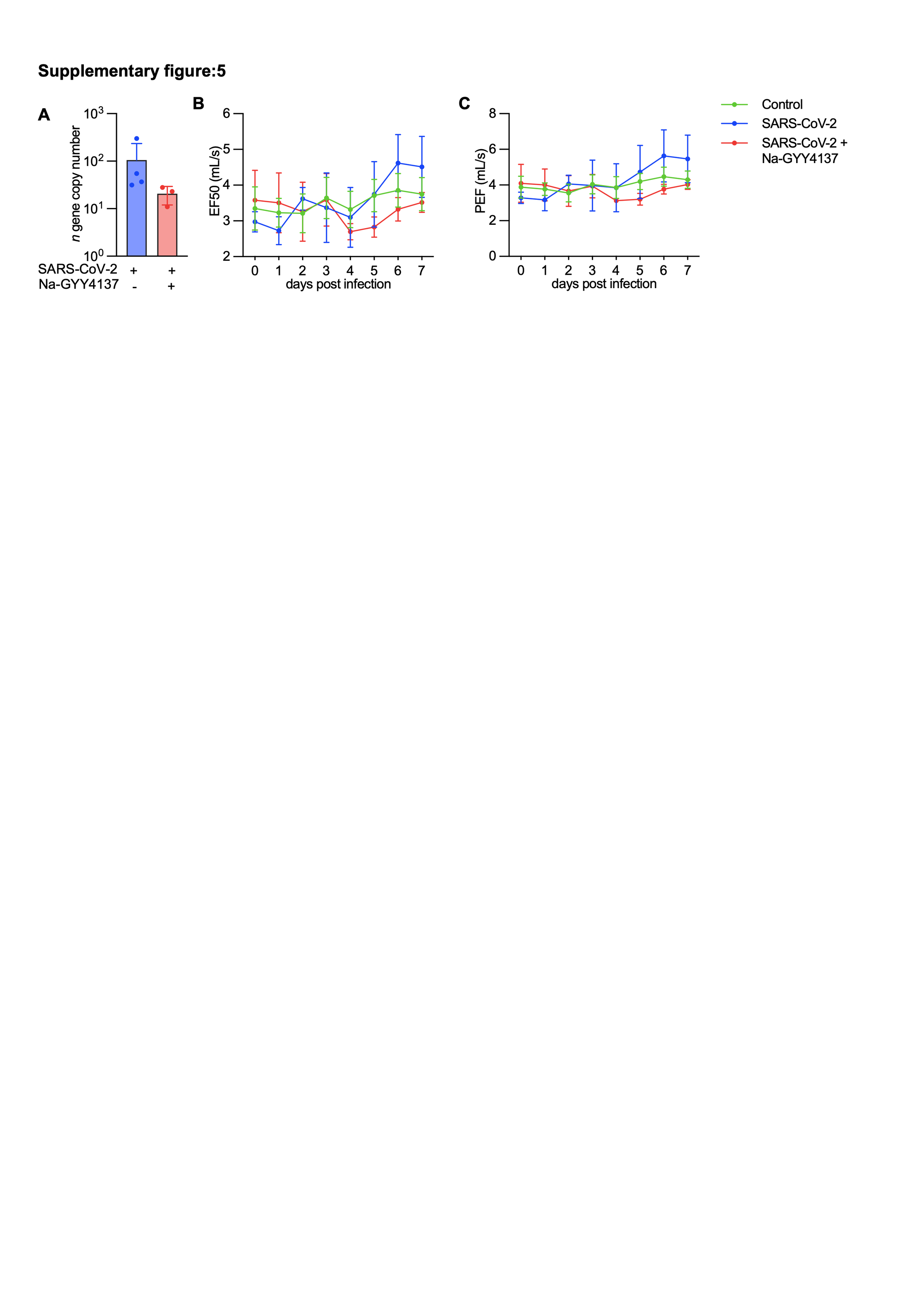

Supplement: S5 Fig — (B) Lung function parameters measured by whole body plethysmography. (TIF) [file ppat.1013164.s005.tif]
