# Supplementary material for: Hydrogen sulfide (H2S) coordinates redox balance, carbon metabolism, and mitochondrial bioenergetics to suppress SARS-CoV-2 infection
Source: PLoS Pathog. 2025 May 19;21(5):e1013164. doi: 10.1371/journal.ppat.1013164 (PMC12129340; doi:10.1371/journal.ppat.1013164)
Supplement: S1 Table — (DOCX) [file ppat.1013164.s006.docx]

| **Gene name** | **Accession number** | **Target Sequence** | **Code** |
| --- | --- | --- | --- |
| *cbs* | NM_000071.2 | GCCGTCAGACCAAGTTGGCAAAGTC | C10 |
| *cth* | NM_001902.4, NM_153742.3 | GGCACCTCATTATCTTTCATAACT | E8 |
| *mst* | NM_021126 | AGAAGAAAGTGGACCTGTCTA | A8 |
| *keap1* | NM_012289.3, NM_203500.1 | CGGGAGTACATCTACATGCAT | A10 |

**Table 1:** List of shRNA constructs used in the study
