# Supplementary material for: Hydrogen sulfide (H2S) coordinates redox balance, carbon metabolism, and mitochondrial bioenergetics to suppress SARS-CoV-2 infection
Source: PLoS Pathog. 2025 May 19;21(5):e1013164. doi: 10.1371/journal.ppat.1013164 (PMC12129340; doi:10.1371/journal.ppat.1013164)
Supplement: S2 Table — (DOCX) [file ppat.1013164.s007.docx]

| **Species** | **Gene** | **Primer** | **Sequence (5'-3')** |
| --- | --- | --- | --- |
| Human | *actin* | Forward | ATGTGGCCGAGGACTTTGATT |
|  |  | Reverse | AGTGGGGTGGCTTTTAGGATG |
|  | *cbs* | Forward | GCCTGAAGTGTGAGCTCTTG |
|  |  | Reverse | CACGATGATGCAGCGATAGC |
|  | *cth* | Forward | TATTTACTCTGGCCGAGAGC |
|  |  | Reverse | TCCTCTAAGCCCACAGAAAG |
|  | *mst* | Forward | GCCGCTTTCTTCGACATC |
|  |  | Reverse | TGGCGTCGTAGATCACG |
|  | *b2m* | Forward | GCCCAAGATAGTTAAGTGGGATCG |
|  |  | Reverse | TCATCCAATCCAAATGCGGC |
|  | *18s* | Forward | GGCCCTGTAATTGGAATGAGTC |
|  |  | Reverse | CGCTCCCAAGATCCAACTAC |
|  | *gclc* | Forward | GGCACAAGGACGTTCTCAAGT |
|  |  | Reverse | CAAGGGTAGGATGGTTTGGG |
|  | *ho-1* | Forward | TAGAAGAGGCCAAGACTGCG |
|  |  | Reverse | GGGCAGAATCTTGCACTTTGTT |
|  | *txn* | Forward | CCCTCTCTGAAAAGTATTCCAACG |
|  |  | Reverse | TGGCTCCAGAAAATTCACCCA |
|  | *txnrd-1* | Forward | ATGGGCAATTTATTGGTCCTCAC |
|  |  | Reverse | CCCAAGTAACGTGGTCTTTCAC |
|  | *cat* | Forward | TTAATCCATTCGATCTCACC |
|  |  | Reverse | GGCGGTGAGTGTCAGGATAG |
|  | *gsr* | Forward | CTTGCGTGAATGTTGGATGT |
|  |  | Reverse | GACCTCTATTGTGGGCTTGG |
|  | *gpx-4* | Forward | GCCTTCCCGTGTAACCAGT |
|  |  | Reverse | GCGAACTCTTTGATCTCTTCGT |
|  | *gpx-1* | Forward | CAACCAGTTTGGGCATCAG |
|  |  | Reverse | GTTCACCTCGCACTTCTCG |
|  | *ace-2* | Forward | TCCATTGGTCTTCTGTCACC |
|  |  | Reverse | AGACCATCCACCTCCACTTCTC |
|  | *tmprss2* | Forward | CCTCTAACTGGTGTGATGGCGT |
|  |  | Reverse | TGCCAGGACTTCCTCTGAGATG |
| Mouse | *gpx-1* | Forward | GGTTCGAGCCCAATTTTACA |
|  |  | Reverse | CCCACCAGGAACTTCTCAAA |
|  | *gpx-4* | Forward | ACGTCAGTTTTGCCTCATTG |
|  |  | Reverse | CTCCATGCACGAATTCTCAG |
|  | *cat* | Forward | GGACGCTCAGCTTTTCATTC |
|  |  | Reverse | TTGTCCAGAAGAGCCTGGAT |
|  | *gclc* | Forward | ACACCTGGATGATGCCAACGAG |
|  |  | Reverse | CCTCCATTGGTCGGAACTCTAC |
|  | *txnrd-1* | Forward | AGTCACATCGGCTCGCTGAACT |
|  |  | Reverse | GATGAGGAACCGCTCTGCTGAA |
|  | *tnfa* | Forward | CTGCACTTTGGAGTGATCGG |
|  |  | Reverse | TCAGCTTGAGGGTTTGCTAC |
|  | *il-6* | Forward | TACCACTTCACAAGTCGGAGGC |
|  |  | Reverse | CTGCAAGTGCATCATCGTTGTTC |
|  | *il-12* | Forward | TTTATGATGGCCCTGTGCCT |
|  |  | Reverse | CAGCTCATCAATAACTGCCAGC |
|  | *b2m* | Forward | ATTCACCCCCACTGAGACTG |
|  |  | Reverse | TGCTATTTCTTTCTGCGTC |
| Viral | *n* | Forward | CACATTGGCACCCGCAATC |
|  |  | Reverse | GAGGAACGAGAAGAGGCTTG |

**Table 2:** List of primers used in the study
