# Supplementary material for: Hydrogen sulfide (H2S) coordinates redox balance, carbon metabolism, and mitochondrial bioenergetics to suppress SARS-CoV-2 infection
Source: PLoS Pathog. 2025 May 19;21(5):e1013164. doi: 10.1371/journal.ppat.1013164 (PMC12129340; doi:10.1371/journal.ppat.1013164)
Supplement: Attachment — Submitted filename: Response to reviewers.pdf [file ppat.1013164.s010.pdf]

PPATHOGENS-D-25-00105

Dear Editors,

We thank the reviewers for responding enthusiastically to our manuscript " Hydrogen sulfide (H_2S) coordinates redox balance, carbon metabolism, and mitochondrial bioenergetics to suppress SARS-CoV-2 infection". We also thank them for providing thoughtful comments, which we have addressed in the revised manuscript. Below, we provide point-by-point responses to the reviewers' comments.

Part I-Summary

Please use this section to discuss strengths/weaknesses of study, novelty/significance, general execution and scholarship.

Reviewer #1: Ragini Agrawal et al. demonstrate that H2S gas participates in the modulation of SARS-CoV-2 infection by suppressing virus replication. The down-regulation of H2S-producing enzymes, including CBS, CTH, and 3-MST, has been observed to be associated with virus replication. The use of GYY4137, a slow-releasing H2S donor, for chemical supplementation of H2S was found to diminish virus replication. This was achieved by inducing the Nrf2/Keap1 pathway, restoring redox balance and carbon metabolites, and potentiating mitochondrial oxidative phosphorylation.
This study is well-written and nicely organized and presented, with carefully crafted figures. However, I believe there are a number of minor to moderate changes that could be introduced to improve the manuscript.

Reviewer #2: In this manuscript, the authors investigate the role of H2S on SARS-CoV-2 replication. Previous research from this group and others has demonstrated the antiviral properties of H2S on respiratory pathogens. The authors conclude that SARS-CoV-2 limits intracellular H2S in infected cells and H2S inhibits viral replication through modulation of the metabolic pathways. Further increasing H2S through pharmalogical intervention blunted viral replication in the lung of infected mice and hamsters, leading to reduced pathology and respiratory distress.

This is a very well written manuscript with several different lines of evidence that support the authors conclusion. I have noted below one alternative hypothesis that should be addressed.

We thank the reviewers for nicely summarising the work and recognizing our efforts in providing a comprehensive study on the role of H_2S in SARS-CoV-2 infection.

Part II – Major Issues: Key Experiments Required for Acceptance
Please use this section to detail the key new experiments or modifications of existing experiments that should be absolutely required to validate study conclusions.

Generally, there should be no more than 3 such required experiments or major modifications for a "Major Revision" recommendation. If more than 3 experiments are necessary to validate the study conclusions, then you are encouraged to recommend "Reject".

Reviewer #1: 1.Previous papers (PMID: 32515982) have shown that H2S may block SARS-CoV-2 entry into host cells by interfering with ACE2 and TMPRSS2, inhibit SARS-CoV-2 replication by attenuating virus assembly/release, and protect SARS-CoV-2-induced lung damage by suppressing immune response and inflammation development. I suggest the authors to detect the expression of ACE2 and TMPRSS2 in the paper to exclude its function in the decreased virus replication.

Response: We thank the reviewers for this comment. In the publication cited by the reviewer [1], the authors raised the possibility of H_2S inhibiting SARS-CoV-2 entry and multiplication by regulating the expression of ACE2 and TMPRSS2. This assumption is based on studies in the mouse/rat models of atherosclerosis and hypertension wherein H_2S treatment significantly altered ACE2 expression in cardiac and kidney tissues [2,3]. Furthermore, studies in epithelium-derived human prostate cancer cells showed marked repression of TMPRSS2 transcription upon H_2S treatment [4]. Based on the reviewer's suggestion, we examined the expression of *ace2* and *tmprss2* in VeroE6 cells by RT-qPCR. As shown in Fig. 1A [Supplementary Fig. 2F in the revised manuscript], SARS-CoV-2 infection uniformly suppresses *ace2* expression in an MOI and time-dependent manner in VeroE6 cells. However, treatment with GYY4137 did not influence *ace2* expression in the SARS-CoV-2 infected or uninfected VeroE6 cells (Fig. 1B and 1C; [Supplementary Fig. 2F in the revised manuscript]). The lack of influence of GYY4137 on *ace2* expression is consistent with studies in BEAS-2B and Calu-1 cells infected with SARS-CoV-2 [5]. TMPRSS2 expression is absent from VeroE6 cells [6]. Consistent with this, our RT-qPCR did not detect *tmprss2* expression in VeroE6 cells, precluding the role of TMPRSS2 in H_2S mediated SARS-CoV-2 inhibition in VeroE6 cells. We further verified these findings in Calu-3 cells. Consistent with VeroE6 cells, we observed a marginal down-regulation of *ace2* and *tmprss2* expression upon SARS-CoV-2 infection. The observed downregulation was maintained even when the infected Calu-3 cells were treated with GYY4137(Fig. 1D [Supplementary Fig. 2G in the revised manuscript]). Taken together, although we cannot entirely exclude the roles ACE2 and TMPRSS2 in H_2S-mediated inhibition of SARS-CoV-2, in our experimental setup, the expression of these receptors is not affected by GYY4137. We have discussed the same in the revised manuscript [Manuscript line 201-211, Supplementary Fig. 2F-G].

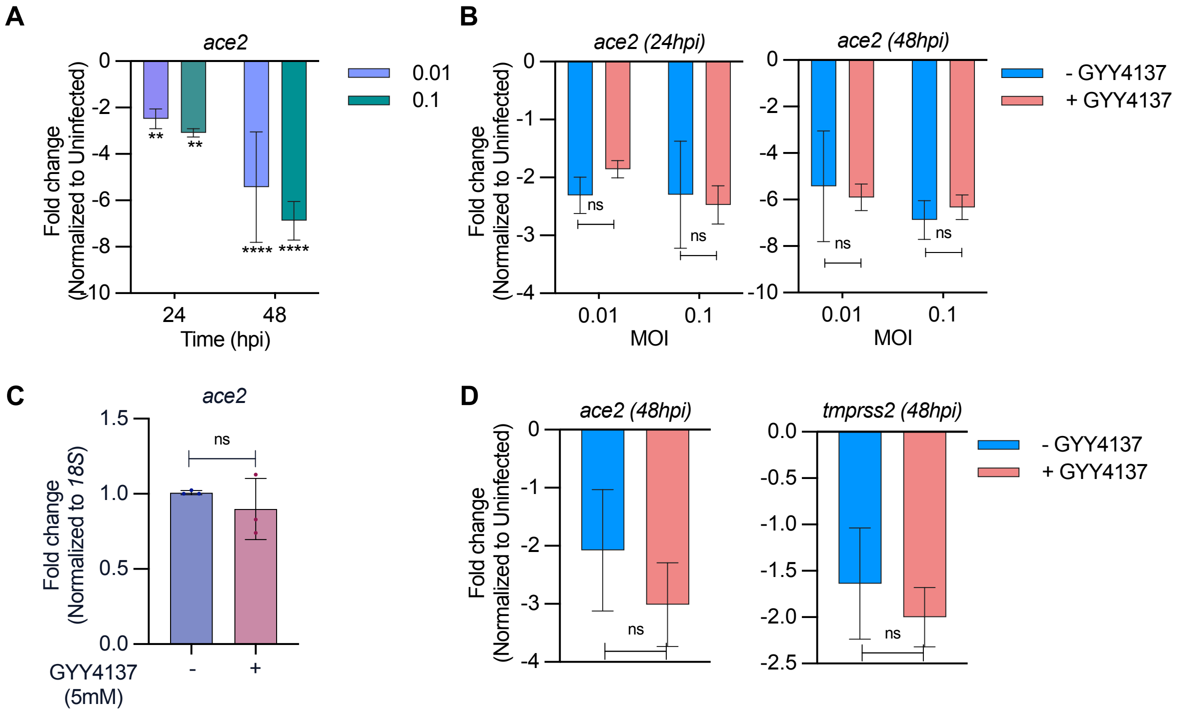

Figure 1: SARS-CoV-2 replication reduces *ace2* expression: (A) VeroE6 cells infected with SARS-CoV-2 at an MOI of 0.01 and 0.1. Infected cells were harvested at 24 and 48 h p.i. to measure *ace2* expression by qRT-PCR. (B) *ace2* expression was measured in VeroE6 cells infected with 0.01 and 0.1 MOI SARS-CoV-2 in presence and absence of 5 mM GYY4137. (C) *ace2* expression in VeroE6 cells treated with or without 5 mM GYY4137. (D) *ace2* and *tmprss2* expression in Calu-3 cells, infected with 0.1 MOI SARS-COV-2 in presence or absence of 5 mM GYY4137 at 48 h p.i.

2.In Fig 5I: "We noticed that treatment with GYY4137 specifically reversed the effect of SARS-CoV-2-HK infection on the expression of genes associated with the oxidoreductase complex and Nrf2-antioxidant pathway (Fig. 3I)". Were the oxidoreductase complex also analyzed in the transcriptional data?

Response: We apologize for the lack of clarity. The genes associated with oxidoreductase activity (*mthfr, acads, sc5d, hsd17b7, cbr4, msmo1, cat, g6pd, nos3, mgst1, sqle, hibadh, and me1)* were downregulated upon SARS-CoV-2, and GYY4137 treatment reduces the extent of down-regulation in our RNA-seq data. To clarify this, we have included the heatmap of genes associated with the oxidoreductase activity in the supplementary information (Fig. 2; [Supplementary Fig. 3B in the revised manuscript, Manuscript line 297-299]).

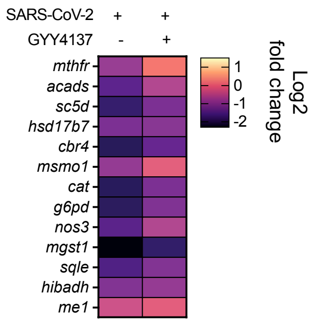

Figure 2: Heat maps of genes associated with oxidoreductase activity upon SARS-CoV-2 infection in presence or absence of GYY4137 with respect to uninfected VeroE6 cells.

3.The full dataset of the RNA-seq needs to be provided in the supplemental information.

Response: We have added the full dataset (normalized CPM value files) in the supplemental information [Supplementary sheet 1, Manuscript line 1438].

4.In line 247-250: "Consistent with this finding, we observed down-regulation of antioxidant genes (e.g., glutathione peroxidases [gpx4], peroxiredoxins [prdx6], hemoxygenase (hmox1], catalase [cat], and thioredoxins [txnrd1])) regulated by nuclear factor erythroid 2-related factor 2 (Nrf2)-a central controller of cellular resistance to redox stress[34]". This concluded from the experiment or Reference 34? In addition, I could find prdx6 in the results throughout the manuscript.

Response: We apologize for the lack of clarity. Our findings were from the RNA seq and qRT-PCR data (Fig 3D and J). We included reference 34 as a reference that the down-regulated genes are the part of Nrf2 regulon. "There is an inadvertent error regarding prdx6, we meant prdx5, not prdx6. We have corrected the error in the revised manuscript [Manuscript line 273, 275].

5.In line 255-257: “As with our RT-qPCR data, infection with SARS-CoV-2 reduces the expression of genes associated with sulfur metabolism, which include H2S biogenesis (Fig. 3D).” The results presented in Figure 3D are inconsistent with the description.

Response: We agree with the reviewer. The confusion is due to the segregation of sulfur metabolism genes into two overlapping categories (sulfur metabolism, and cysteine-methionine metabolism). We have now unified this into one functional category of sulfur metabolism (Fig. 3; [Fig 3D in the revised manuscript]). Our RNA-seq data on H_2S biogenesis genes (*mst*, *cth*) agrees with our RT-qPCR data (Fig 1 C and E; [Supplementary figure 1 A and B] in the revised manuscript).

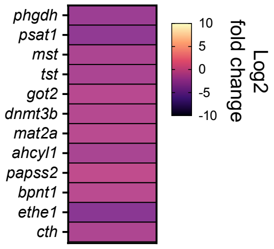

Figure 3: Heat maps of genes associated with sulfur metabolism upon SARS-CoV-2 infection compared to uninfected VeroE6 cells.

6.The authors should clarify why the Nrf2 gene was not included in the RNA-seq data, despite its expression being detected by WB.

Response: Nrf2 (encoded by *nfe2l2*) was not featured in our RNA-seq data because neither infection nor GYY4137 treatment affected the expression of *nfe2l2* transcript (Fig 4). Our observation is consistent with the fact that the expression of Nrf2 is regulated post-translationally. Under the basal conditions, Nrf2 levels are controlled by Keap1, which acts as an adaptor molecule for Nrf2 and E3 ubiquitin ligase, and targets Nrf2 for proteasomal degradation. In response to stress, stabilization of Nrf2 is thought to be dependent on mechanisms that either prevent or reduce access of Keap1 to Nrf2 resulting in the downregulation of NRF2 protein levels without affecting its transcription [7]. We have included this information in the revised manuscript [Supplementary Fig. 3A, Manuscript line 277-279].

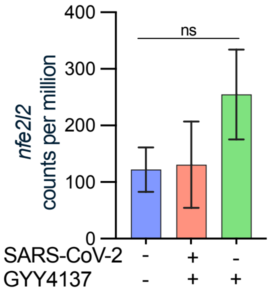

Figure 4: *nfe2l2* transcript (counts per million) upon SARS-CoV-2 infection in absence and presence of GYY4137 by RNA sequencing.

7.According to the results, H2S suppression of SARS-CoV-2 proliferation is associated with the expression of the Nrf2/Keap-1 regulon, redox metabolites, and mitochondrial function. Therefore, what is the relationship among these factors? Which one serves as the primary influencing factor?

Response: We appreciate the reviewer's question on the primary vs secondary influence of H_2S on Nrf2/Keap-1 regulon, redox metabolites, and mitochondrial function. H_2S is known to directly modulate mitochondrial activity and redox balance via multiple mechanisms including Nrf2/Keap1 axis [7]. Although the primary vs secondary effect of H_2S is challenging to resolve, our RNA-seq data points toward the important role of Nrf2/Keap1 as an upstream mediator of H_2S signalling on SARS-CoV2 infection. H_2S is shown to directly persulfidate the Keap1 at cysteine residues, which structurally modifies Keap1 to induce Nrf2 nuclear translocation and stimulates the expression of antioxidant genes [8]. Therefore, Keap-1 functions as one of the intracellular sensors of H_2S. We bolster the primary role of Nrf2/Keap-1 by depleting the endogenous levels of Keap1 in VeroE6 cells and showing that the effect of GYY4137 was significantly reduced (~35 fold) in Keap1 knockdown cells (Fig. 5G in the revised manuscript). Additionally, H_2S-mediated S-persulfidation is reversible upon treatment of cells with a thiol reductant (DTT) [8,9]. Consistent with this, we showed that the virus-suppressing effect of GYY4137 was significantly reversed by treating VeroE6 cells with DTT (Fig. 5H in the revised manuscript). Taken together, data suggest that H_2S likely prevents virus replication by activating the Nrf2 via Keap1 persulfidation, which results in restoring redox balance and mitochondrial function. We have included these explanations in the revised manuscript [Manuscript line 521-530]. We thank the reviewer for this comment.

8.The authors should clarify the rationale behind using different treatments in Figure 3 and Figure 4. Additionally, they should discuss whether this discrepancy in treatment affects the results obtained.

Response: We thank the reviewers for highlighting this issue. Figure 3 pertains to RNA-seq analysis of VeroE6 cells in response to SARS-CoV-2 infection and GYY4137 treatment. In these experiments, we used a higher concentration of GYY4137 as the lower concentration had no influence on gene expression. For example, infection with SARS-CoV-2 resulted in downregulating the *nrf2* regulon, which was restored upon treatment with 5 mM GYY4137 but not with 10 μM GYY4137 (Fig 5). In contrast to RNA-seq, metabolomic changes documented in figure 4 were observed at a lower concentration of GYY4137 (10 μM). The corresponding intensities of metabolites were not uniformly detected at higher concentrations of GYY4137. Since we have measured steady-state levels of metabolites in response to GYY4137 treatment, it is challenging to ascertain dynamic changes in metabolites in response to variations in GYY4137 treatment. To accurately associate metabolite changes with the GYY4137 concentrations, a flux-based analysis involving the pulse-label of a metabolite and the estimate of label incorporation in the metabolic products is needed since the steady-state amounts cannot separate production from consumption. Therefore, it will be difficult to compare RNA-seq data that show dynamic changes in the transcript levels due to gene regulation with the steady-state amounts of the metabolites. Future flux-based experimentations are needed to correlate transcriptome with the metabolomic changes. This will be a part of an independent study.

However, in light of the reviewer's comment, we appropriately discussed the dosing effects of H_2S donors (Line 560-574 in the revised manuscript). The higher concentration of H_2S is associated with toxicity and side effects, whereas lower micromolar concentrations are beneficial [10]. However, the intracellular concentration of H_2S in response to donors will

depend upon absorption and metabolism, which are reasonably well characterized. The sulfide oxidation pathways are involved in the oxidation of excess H_2S to thiosulphate and sulphate, which are readily cleared from the body via the urine in free or conjugated form [10]. S-adenosyl methionine (SAM)-dependent methylation of H_2S by human methyltransferase-like protein 7B (METTL7B) could be another mechanism to reduce potential toxicity [11]. All of these observations would have led to successful application of high doses of GYY4137/NA-GYY4137 in suppressing virus proliferation and lung pathology in mice and hamsters. We assessed the cytotoxicity of the H_2S donor (GYY4137) in all cell lines and selected non-toxic concentrations of GYY4137 to perform cell-based assays. Animal experiments were based on published safe doses of GYY4137 [12].

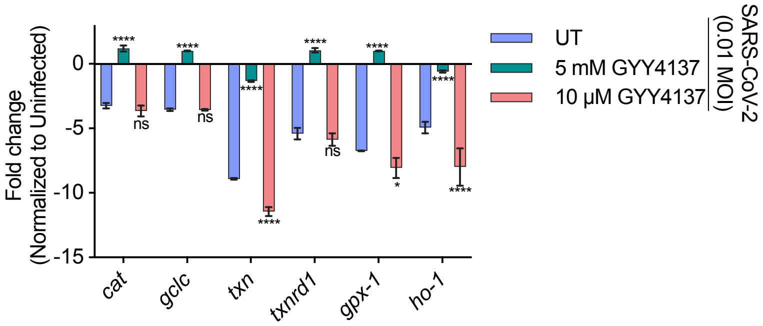

Figure 5: Nrf2 dependent gene expression upon SARS-CoV-2 infection in presence or absence of GYY4137.

Reviewer #2: The authors conclude that H2S suppresses viral replication through modulation of host cell metabolic pathways. However an alternative conclusion from the data could be that H2S inhibits viral entry and cells are less infected. All of the experiments in this manuscript pretreat the cells/mice with higher levels of H2S, either through treatment with a slow H2S release chemical or genetic manipulation of the H2S synthesis pathways. To address this alternative hypothesis, I suggest the following experiment. Infect cells at a high MOI (3-5). Wash cells after one hour and treat with H2S. Monitor viral growth kinetics.

Response: We agree with the reviewer that it is important to examine alternative hypotheses. As suggested by the reviewer, we infected VeroE6 cells with SARS-CoV-2 at a high MOI of 3 for 1 h, washed the cells, and treated them with the H_2S donor GYY4137 post-infection (Post-entry). We measured viral growth at an early time point (8 h post-infection) to assess the contribution of defective entry. As a control, we repeated the experiment wherein cells were pre-treated with GYY4137 and continued the treatment for 8 hours (Full-time). The expression of *n* gene was assessed by RT-qPCR. As expected, pre-treatment resulted in ~ 300-fold reduction in *n* gene transcript compared to untreated samples. Importantly, post-infection treatment with GYY4137 also resulted in a significant decrease (~40-fold) in *n* gene copy number than control (Fig 6, Supplementary Fig. 2H in the revised manuscript). Therefore it appears that GYY4137 inhibits virus proliferation by targeting entry and post-entry steps. As an additional verification, we co-incubated VeroE6 cells with GYY4137 and SARS-CoV-2 virus for 1 h, washed virus and GYY4137, and measured *n* gene expression at 8 h p.i (During entry). As shown in Fig. 6 (Supplementary Fig. 2H in the revised manuscript), an ~ 80-fold reduction in the *n* gene expression compared to untreated control further indicates the role of mechanisms associated with virus entry in H_2S-mediated suppression of SARS-CoV-2. Future experimentations are needed to comprehensively understand the relative contribution of pre-entry and post-entry mechanisms modulated by GYY4137 to inhibit SARS-CoV-2. We have

included the data in the revised manuscript [Manuscript line 211-224, 546-548, Supplementary Fig. 2H]. We thank the reviewer for this excellent recommendation.

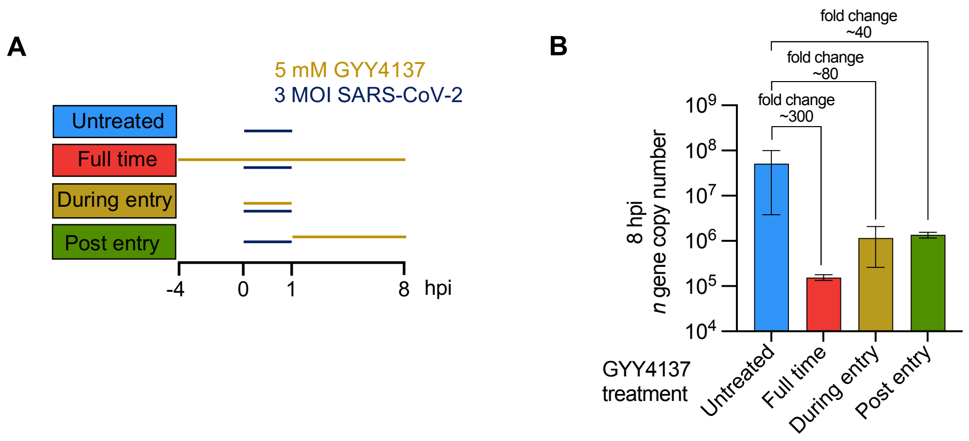

Figure 6: Time of addition experiment: (A) Schematic of the experiment. Briefly, VeroE6 cells either pre-treated with 5 mM GYY4137 or left untreated, followed by infection with SARS-CoV-2 at an MOI of 3 for 1 h in presence or absence of GYY4137. Post infection, cells were either treated or left untreated with 5 mM GYY4137. (B) Viral titre measured at 8 h p.i. by RT-qPCR.

Part III – Minor Issues: Editorial and Data Presentation Modifications
Please use this section for editorial suggestions as well as relatively minor modifications of existing data that would enhance clarity.

Reviewer #1: 1.Fig. 2A: The label for Fig. 2A is missing in the Results section.

Response: In the revised manuscript, we have added the label for Fig 2A in results section [Manuscript line 157, 166, 175].

2.In line 225-232: I was unable to locate the genes in Fig 3, and the reference was not provided either. Kindly provide a detailed description of it.

Response: In the revised manuscript, we have modified the Fig 3 for the gene expression data [Manuscript figure 3C].

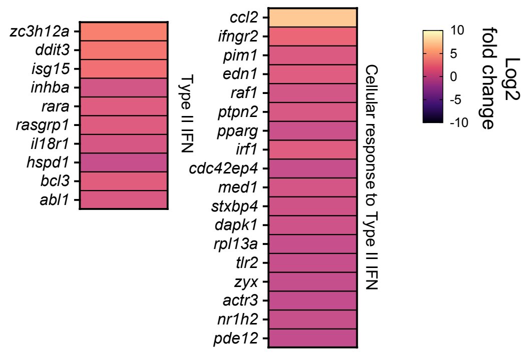

Figure 7: Gene response to type II interferon production and cellular response to type II interferon upon SARS-CoV-2 infection.

3.Fig. 6I: Based on the text, there should be a label (7) missing in Fig. 6I. Furthermore, please clarify the meaning of 'BV' in Fig. 6I?

Response: The label (7) is for Fig 6C. To avoid confusion, in the revised manuscript we have modified the legend. BV denotes Blood vessels. It has been added in the revised manuscript [Manuscript line 1399, 1403].

4.In lines 1314-1315: "(B) denotes bronchial lumen and (AS) denotes alveolar space". These annotations may require revision.

Response: In the revised manuscript we have modified the annotations as follow: Bronchial lumen (BL)[Manuscript line 1403, Manuscript figure 6C].

Reviewer #2: - Fig 1H. Violin plots are distracting. Please show scatter plot of data points.

Response: In the revised manuscript we have modified the violin plot to the scatter plot [Manuscript figure 1H].

- Fig 6B right panel. Y-axis title should read n gene copy number. Also how is this data normalized?

Response: The Y-axis title in Fig 6B is correct. For this assay, we used a Taqman-based kit for the detection of viral *e* gene (Q-line-ER (nCoV-19)RT-PCR detection kit). An equal concentration of RNA was used for RT-qPCR. We generate a standard curve using known viral titre to calculate the copy number of the *e* transcript. We have added the detailed protocol in the methods section in the revised manuscript [Manuscript line 841-844]. We thank the reviewer for this comment.

- Fig 6 general: Make groups across different panels the same color. For example SARS-CoV-2 + GYY4137 is red in 6A-E, then green in 6F-H.

Response: The same has been rectified in the revised manuscript [Manuscript Fig 6 F-H, Supplementary figure 5 A-C].

References:

1. Yang G. H2S as a potential defense against COVID-19? Am J Physiol Cell Physiol. 2020;319: C244–C249. doi:10.1152/ajpcell.00187.2020

2. Lin Y, Zeng H, Gao L, Gu T, Wang C, Zhang H. Hydrogen Sulfide Attenuates Atherosclerosis in a Partially Ligated Carotid Artery Mouse model via Regulating Angiotensin Converting Enzyme 2 Expression. Front Physiol. 2017;8: 782. doi:10.3389/fphys.2017.00782

3. Tain Y-L, Hsu C-N, Lu P-C. Early short-term treatment with exogenous hydrogen sulfide postpones the transition from prehypertension to hypertension in spontaneously

hypertensive rat. Clin Exp Hypertens N Y N 1993. 2018;40: 58–64. doi:10.1080/10641963.2017.1313847

4. Zhao K, Li S, Wu L, Lai C, Yang G. Hydrogen sulfide represses androgen receptor transactivation by targeting at the second zinc finger module. J Biol Chem. 2014;289: 20824–20835. doi:10.1074/jbc.M114.559518

5. Pozzi G, Masselli E, Gobbi G, Mirandola P, Taborda-Barata L, Ampollini L, et al. Hydrogen Sulfide Inhibits TMPRSS2 in Human Airway Epithelial Cells: Implications for SARS-CoV-2 Infection. Biomedicines. 2021;9: 1273. doi:10.3390/biomedicines9091273

6. Matsuyama S, Nao N, Shirato K, Kawase M, Saito S, Takayama I, et al. Enhanced isolation of SARS-CoV-2 by TMPRSS2-expressing cells. Proc Natl Acad Sci U S A. 2020;117: 7001–7003. doi:10.1073/pnas.2002589117

7. Ma Q. Role of Nrf2 in Oxidative Stress and Toxicity. Annu Rev Pharmacol Toxicol. 2013;53: 401–426. doi:10.1146/annurev-pharmtox-011112-140320

8. Xie L, Gu Y, Wen M, Zhao S, Wang W, Ma Y, et al. Hydrogen Sulfide Induces Keap1 S-sulfhydration and Suppresses Diabetes-Accelerated Atherosclerosis via Nrf2 Activation. Diabetes. 2016;65: 3171–3184. doi:10.2337/db16-0020

9. Yang C, Devarie-Baez NO, Hamsath A, Fu X, Xian M. S-Persulfidation: Chemistry, Chemical Biology, and Significance in Health and Disease. Antioxid Redox Signal. 2020;33: 1092–1114. doi:10.1089/ars.2019.7889

10. Kabil O, Banerjee R. Redox Biochemistry of Hydrogen Sulfide*. J Biol Chem. 2010;285: 21903–21907. doi:10.1074/jbc.R110.128363

11. Maldonato BJ, Russell DA, Totah RA. Human METTL7B is an alkyl thiol methyltransferase that metabolizes hydrogen sulfide and captopril. Sci Rep. 2021;11: 4857. doi:10.1038/s41598-021-84218-5

12. Ivanciuc T, Sbrana E, Ansar M, Bazhanov N, Szabo C, Casola A, et al. Hydrogen Sulfide Is an Antiviral and Antiinflammatory Endogenous Gasotransmitter in the Airways. Role in Respiratory Syncytial Virus Infection. Am J Respir Cell Mol Biol. 2016;55: 684–696. doi:10.1165/rcmb.2015-0385OC
